# Parental Drought-Priming Enhances Tolerance to Post-anthesis Drought in Offspring of Wheat

**DOI:** 10.3389/fpls.2018.00261

**Published:** 2018-03-01

**Authors:** Xiulin Wang, Xiaxiang Zhang, Jing Chen, Xiao Wang, Jian Cai, Qin Zhou, Tingbo Dai, Weixing Cao, Dong Jiang

**Affiliations:** National Technique Innovation Center for Regional Wheat Production, Key Laboratory of Crop Physiology and Ecology in Southern China, Ministry of Agriculture, National Engineering and Technology Center for Information Agriculture, Nanjing Agricultural University, Nanjing, China

**Keywords:** drought priming, drought stress tolerance, photosynthesis, osmolytes, antioxidant capacity, winter wheat (*Triticum aestivum* L.)

## Abstract

Drought is the major abiotic stress that decreases plant water status, inhibits photosynthesis, induces oxidative stress, restricts growth and finally lead to the reduction of wheat yield. It has been proven that drought priming during vegetative growth stage could enhance tolerance to drought stress at grain filling in wheat. However, whether drought priming imposed at grain filling in parental plants could induce drought tolerance in the offspring is not known. In this study, drought priming was successively applied in the first, the second and the third generation of wheat to obtain the plants of T1 (primed for one generation), T2 (primed for two generations), T3 (primed for three generations). The differently primed plants were then subjected to drought stress during grain filling in the fourth generation. Under drought stress, the parentally primed (T1D, T2D, T3D) plants, disregarding the number of generations, showed higher grain yield, leaf photosynthetic rate and antioxidant capacity as well as lower $O_{2}^{•-}$ release rate and contents of H_2_O_2_ and MDA than the non-primed (T0D) plants, suggesting that drought priming induced the transgenerational stress tolerance to drought stress. Moreover, the parentally primed plants showed higher leaf water status, which may result from the higher contents of proline and glycine betaine, and higher activities of Δ1-pyrroline-5-carboxylate synthetase (P5CS) and betaine aldehyde dehydrogenase (BADH), compared with the non-primed plants under drought stress. In addition, there was no significant difference among three generations under drought, and the drought priming in parental generations did not affect the grain yield of the offspring plants under control condition. Collectively, the enhanced accumulation of proline and glycine betaine in the parentally primed plants could have played critical roles in parental priming induced tolerance to drought stress. This research provided a potential approach to improve drought tolerance of offspring plants by priming parental plants.

## Introduction

Drought is one of the critical environmental adversities affecting the growth, development and final yield of crop species (Geng et al., 2016; Daryanto et al., 2017), and the frequency and severity of drought stress events are expecting to increase due to global climate change (Cook et al., 2014; Zhao and Dai, 2015; Joshi et al., 2016). Drought stress perturbs a broad range of plant physiological and biochemical processes, including decreased plant water status, inhibited photosynthetic processes, induced oxidative stress damage and so on, which ultimately lead to growth retardation and the reduction of crop yield (Perdomo et al., 2015; Saeidi and Abdoli, 2015; Daryanto et al., 2017). Wheat, one of the major food crop, is susceptible to drought stress especially during grain filling, which is the most critical stage determining the final grain weight (Yang and Zhang, 2006). Therefore, improving plant tolerance to drought stress occurring during grain filling in wheat is meaningful for sustaining food security under the future climate.

Drought stress happened during grain filling could reduce wheat yield significantly, while drought tolerant varieties could maintain lower grain yield loss than the drought sensitive varieties (Saeedipour and Moradi, 2011). Such yield loss was largely due to the inhibition of photosynthesis under drought (Austin et al., 1977; Huseynova et al., 2016). The inhibition of plant photosynthesis may be resulted from the stomatal and/or non-stomatal limitation, which depended on the severity of drought stress (Caemmerer and Farquhar, 1981; Lawlor and Tezara, 2009). In addition, the reactive oxygen species (ROS) burst under severe drought could cause oxidative stress damage, resulting in disturbance of a series of physiological and biochemical processes (Cruz de Carvalho, 2008). Morphologically, plants under drought reduce the leaf area, decrease the stomatal conductance to reduce the water transpiration, and increase the root distribution in the deeper soil to maximum the water uptake (Farooq et al., 2009; Fang et al., 2017). Besides, plant has developed a series of strategies at physiological, biochemical, and molecular levels to cope with drought stress, such as ABA content increase, ROS removement, osmotic adjustment and gene expression (Chaves et al., 2003). The activation of antioxidant systems, which including enzymatic and non-enzymatic antioxidants, to remove excess ROS is an important strategy to cope with drought stress in plants (Apel and Hirt, 2004). Antioxidant enzymes mainly include superoxide dismutase (SOD), catalase (CAT), ascorbate peroxidase (APX) and glutathione peroxidase (GPX), and non-enzymatic antioxidants mainly include reduced ascorbate (AsA) and reduced glutathione (GSH) (Apel and Hirt, 2004; Miller et al., 2010). Another important biochemical response to drought is osmotic adjustment such as the accumulation of proline and glycine betaine (GB), which can help plants to retain or absorb more water by decreasing the osmotic potential of plant cells, buffer cellular redox potential and maintain the structure and physiological function of biological macromolecules (Hare et al., 1998; Sakamoto and Murata, 2002; Ashraf and Foolad, 2007; Szabados and Savoure, 2010; Wang et al., 2014a).

Several studies have shown that priming (pre-exposure to a moderate stress) could enhance tolerance to subsequent stresses, which is known as the term of “stress memory” (Bruce et al., 2007; Chinnusamy and Zhu, 2009; Slaughter et al., 2012; Pastor et al., 2013). The stress-induced signaling chemicals, proteins, RNAs and metabolites were considered as short term memory factors (Chinnusamy and Zhu, 2009), while epigenetic modifications such as DNA methylation and histone modifications are potential mechanisms for long-term, and even transgenerational memory (Chinnusamy and Zhu, 2009; Hauser et al., 2011; Sani et al., 2013; Migicovsky et al., 2014). Our previous studies have found that drought priming during vegetative growth stage could enhance tolerance to freezing at jointing stage (Li et al., 2015), and drought or heat during grain filling in wheat (Wang et al., 2014b,c). In addition, the offspring of the drought primed plants enhanced stress tolerance to post-anthesis high-temperature stress in wheat through improved photosynthesis and induced anti-oxidation capacity (Zhang et al., 2016). Nosalewicz et al. (2016) reported that the intense drought stress has transgenerational effects on root morphology and topology of offspring in spring barley, where the offspring of the drought primed plants showed relatively decreased shoot-to-root ratio and reduced thick roots number, compared to the non-primed progeny under drought stress. It has been found that drought priming in the parental rice plants could induce proline accumulation through up-regulating expression of the proline synthesis genes paralleling with greater DNA demethylation in the offspring plants under drought (Zhang et al., 2013). However, whether drought priming responses exist in the offspring of primed plants in wheat remains unclear.

In this study, we performed drought priming during grain filling in parental plants for three successive generations, and then subjected the respective offspring plants to drought stress during grain filling. Grain yield, photosynthesis, antioxidant system and osmotic adjustment were analyzed to investigate whether parental drought-priming could enhance tolerance to post-anthesis drought in offspring. The following hypotheses were tested: (i) drought priming in parental plants could enhance tolerance to drought stress in the offspring of wheat; and (ii) osmolytes accumulation may play critical roles in drought priming induced transgenerational drought tolerance.

## Materials and Methods

### Experimental Setup

**FIGURE 1:**
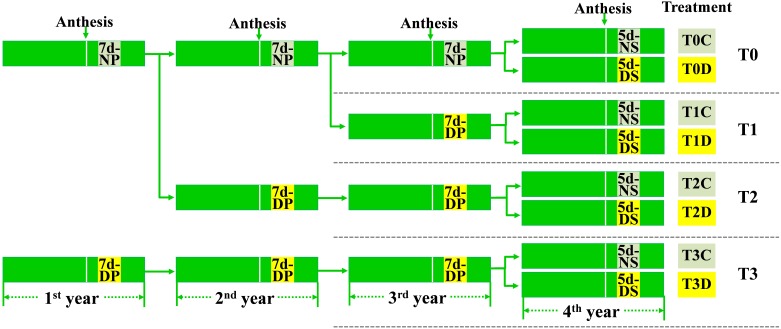
Setup of the experiment. The 7-day drought priming events (NP- no priming, DP- drought priming) were successively applied in the 1st, 2nd, and 3rd growth season (experimental year), the 5-day drought stress (NS, no drought stress; DS, drought stress) was conducted in the 4th year. Both the drought priming and the drought stress were initiated at 10 days after anthesis. In total, eight treatments were given: T0C, no former-generation priming + no offspring drought stress; T0D, no former-generation priming + offspring drought stress; T1C, one-generation priming + no offspring drought stress; T1D, one-generation priming + offspring drought stress; T2C, two-generation priming + no offspring drought stress; T2D, two-generation priming + offspring drought stress; T3C, three-generation priming + no offspring drought stress; T3D, three-generation priming + offspring drought stress.

Drought priming was performed in the cement pools (4 m in length, 2.5 m in width and 0.6 m in depth) with rain-proof shelter as reported in our previous study (Zhang et al., 2016) at the Experimental Station of Nanjing Agricultural University, Nanjing, Jiangsu Province, China. The winter wheat (*Triticum aestivum* L.) cultivar Ningmai 13 was used. As shown in **Figure 1**, the 7-day drought priming event was successively applied in the first, the second and the third growth season (experimental year) to obtain the plants of T1 (primed for one generation), T2 (primed for two generations), T3 (primed for three generations). The plants without drought priming were annotated as T0. Leaf relative water content (LRWC) after drought treatment and the effect of drought on kernel weight of the parental plants were measured (Supplementary Table 1).

The 5-day drought stress was conducted in the fourth year in a rainproof shelter. T0, T1, T2, T3 seeds were grown in plastic pots (22 cm in height and 25 cm in diameter) filled with 7.5 kg of soil (clay soil:sand = 2:1, w/w) with 0.9 g N, 0.36 g P_2_O_5_, and 0.9 g K_2_O mixed per pot. Another 1.6 g N was provided at the jointing stage. Prior to drought treatment, the seedlings were grown with normal water supply. At 10 days after anthesis, half of the T0, T1, T2, T3 seedlings were subjected to drought stress (D) by withholding water until soil relative water content (SRWC) decreased to ca. 40%, and then kept for 5 days before re-watering to the normal level. The SRWC of the rest seedlings maintained at ca. 75% were corresponding control (C). SRWC was measured according to the method of Wang et al. (2014b). Taken together, eight treatments were established as T0D, T0C; T1D, T1C; T2D, T2C; T3D, T3C (**Figure 1**). The experiment was a randomized complete block design, with 30 pots as replicates for each treatment. The flag leaves of each treatment were collected at the third day for gene expression analysis and fifth day for determination of physiological and biochemical indexes during drought stress.

### Plants Water Status

Leaf relative water content (LRWC) of flag leaves was measured as described by Jensen et al. (2000). Predawn water potential of flag leaves (Ψ_w_) was measured with an L-51 leaf hygrometer using HR-33T dew point microvolt-meter (Wescor Inc., Logan, UT, United States). Leaf osmotic potential (Ψ_s_) was determined using a vapor pressure osmometer (Wescor 5600, Wescor Inc., Logan, UT, United States) at 25°C.

### Photosynthesis and Chlorophyll Fluorescence Parameters

Gas exchange of flag leaves was measured on the last day of drought stress using a LI-6400 portable photosynthesis measurement system (LI-COR Biosciences, Lincoln, NE, United States) between 9:00 am and 11:00 am. The CO_2_ concentration in the leaf chamber was set at 380 μmol mol^-1^ and the photosynthetically active radiation (PAR) was set at 1000 μmol m^-2^ s^-1^.

The maximum quantum efficiency of photosystem II (Fv/Fm) after full dark adaptation (30 min) and the actual photochemical efficiency (ΦPSII) were measured with a portable chlorophyll fluorometer PAM-2500 (Heinz Walz GmbH, Eichenring, Effeltrich, Germany). Photochemical quenching (qP) and non-photochemical quenching (NPQ) of chlorophyll fluorescence were calculated according to the description of Maxwell and Johnson (2000).

### Antioxidant System

Content of malondialdehyde (MDA), the final product of lipid peroxidation, was measured as described by Du and Bramlage (1992). $O_{2}^{•-}$ release rate was assayed following hydroxylamine method (Elstner and Heupel, 1976), and H_2_O_2_ content was determined according to the method of Sui et al. (2007). Activities of superoxide dismutase (SOD; EC 1.15.1.1) and catalase (CAT; EC1.11.1.6) were measured according to our previous methods (Tan et al., 2008). Activities of ascorbate peroxidase (APX; EC 1.11.1.11), dehydroascorbate reductase (DHAR; EC 1.8.5.1) and monodehydroascorbate reductase (MDHAR; EC 1.6.5.4) were assayed by the method of Fryer et al. (1998). Activities of glutathione peroxidase (GPX; EC 1.11.1.7) and glutathione reductase (GR; EC 1.6.4.2) were measured using Total Glutathione Peroxidase Assay Kit and Glutathione Reductase Assay Kit from Beyotime institute of Biotechnology (Shanghai, China), respectively. The contents of reduced ascorbate (AsA) and reduced glutathione (GSH) were measured as described by Gossett et al. (1994). The content of soluble protein was measured by the method of Bradford (1976).

### Contents of Proline and GB

Proline content was determined according to ninhydrin coloring method (Bates et al., 1973). 0.1 g of finely ground dried flag leaves was homogenized in 5 ml of 3% aqueous sulfosalicylic acid, heated for 10 min in boiling water bath followed by centrifugation. Two ml of supernatant was reacted with 2 ml of glacial acetic acid and 2 ml of acid-ninhydrin (2.5 g ninhydrin dissolved in 60 ml glacial acetic acid and 40 ml 6 M phosphoric acid) in a test tube for 1 h in boiling water bath. The reaction was terminated in an ice bath. The reaction mixture was mixed with 4 ml toluene completely and then standing for 2 h. The upper layer was used for determining the proline content at 520 nm using a spectrophotometer (UV-1780, Shimadzu (Suzhou) Instruments Manufacturing, Co., Ltd., Suzhou, China).

Glycine betaine (GB) content was measured following the description of Khoshro et al. (2013). Five ml of toluene-water mixture (0.05% toluene) mixed with 0.2 g of finely ground dried flag leaves was mechanically shaken for 24 h at 25°C and then was filtered. Half ml of the filtrate was taken, and 1 ml of 2 N HCl solution, 0.1 ml of potassium tri-iodide solution (100 ml of 1 N HCl containing 7.5 g of iodine and 10 g of potassium iodide) was added. The mixture was incubated in an ice water bath for 90 min. After shaking gently, 10 ml of 1, 2-dichloroethane (chilled at -10°C) was poured into it. Then by passing a continuous stream of air for 2 min, two layers were separated. The absorbance of the organic layer was measured at 365 nm to determine the GB content.

### Activities of Key Enzymes Involving in Proline and GB Metabolism

Extraction of Δ1-pyrroline-5-carboxylate synthetase (P5CS) and proline dehydrogenase (PDH) from wheat flag leaves was conducted according to the description of Tripathi et al. (2013). Half gram of flag leaves were homogenized in 5 ml of 50 mM Tris-HCl buffer (pH 7.4) containing 3 mM EDTANa_2_, 7 mM MgCl_2_, 0.6 mM KCl, 1 mM DTT and 5% (w/v) PVP on ice. Then the homogenate was centrifuged at 4°C for 30 min at 14,000 *g*. The supernatant was used for enzymes activities determination.

P5CS activity was measured according to the method of Tripathi et al. (2013). The reaction mixture volume was 3 ml containing 100 mM Tris-HCl (pH 7.2), 25 mM MgCl_2_, 75 mM sodium glutamate, 5 mM ATP, and 0.2 ml of extract. Then 0.2 ml of 0.4 mM NADPH was added in to initiate the reaction. The decrease in absorbance at 340 nm due to the consumption of NADPH was monitored to calculate P5CS activity.

PDH activity was assayed following the protocol of Tripathi et al. (2013). The total reaction mixture volume was 3.5 ml containing 2.8 ml of 100 mM Na_2_CO_3_-NaHCO_3_ buffer (pH 10.3, contained 20 mM L-proline) and 0.5 ml of enzyme extract, 0.2 ml of 10 mM NAD was finally added to initiate the reaction. The change in absorbance at 340 nm was monitored for calculation of PDH activity.

Betaine aldehyde dehydrogenase (BADH) activity was assayed following the method of Weretilnyk and Hanson (1989). One fifth gram of flag leaves was homogenized in 10 ml of 0.1 M Tricine-KOH buffer (pH 8.5) containing 1 mM EDTANa_2_, 2 mM DTT, and 0.6 M sucrose. The homogenate was centrifuged at 10,000 *g* for 10 min at 4°C and the supernatant was collected. Protein in the supernatant was isolated by solid ammonium sulfate and desalted by centrifugal filtration on Sephadex G-25 columns. Enzyme extract (0.05 ml) was added in 0.95 ml of 0.1 M Tris-HCl buffer (pH 8.0) containing 0.5 mM NAD^+^ and 5 mM DTT, reacted at 30°C for 30 min. BADH activity was measured at 340 nm and represented by NADH production amount per minute.

### RNA Extraction, cDNA Synthesis and Quantitative Real-Time PCR

Total RNA was extracted from 50 to 100 mg of flag leaves using RNAiso Plus reagent (Takara Bio, Japan). The concentration and purity of RNA extract solution were measured with the NanoDrop 2000 (Thermo Scientific, United States) and the integrity was confirmed by agarose gel electrophoresis (1.2%). The removal of residual genomic DNA and first-strand cDNA synthesis was performed using HiScript II Q RT SuperMix for qPCR (+gDNA wiper) (Vazyme Bio, China) according to the instruction.

Sequences and its source of all primers were listed in Supplementary Table 2. Quantitative Real-time PCR was performed using ChamQ SYBR qPCR Master Mix (Vazyme Bio, China) on CFX Connect Real-Time PCR Detection System (Bio-Rad, United States) with cycling parameter: 95°C for 30 s; 40 cycles of 95°C for 10 s, 60°C for 30 s. Melting curves were run after PCR cycles. The relative expression levels of genes were calculated according to the 2^-ΔΔ*Ct*^ method, using *ADP-RF* gene as the reference gene. Three biological repeats and three technical repeats were performed.

ΔΔCt = (Ct target gene – Ct reference gene) treatment – (Ct target gene – Ct reference gene)T0C

### Statistical Analysis

All data presented is the mean ± SE of three independent measurements. Data collected were analyzed using One-way ANOVA by SPSS package Ver. 22.0 (SPSS Inc., Chicago, IL, United States). Duncan’s multiple range test was used to determine significance differences among treatments (*P* < 0.05).

## Results

### Grain Yield and Yield Components

Grain yield was significantly reduced by drought, which was ascribed to the decrease in 1000-kernal weigh rather than number of ears and grain number per ear (**Table 1**). However, 1000-kernal weight yield of T1D (10.26%), T2D (12.02%), T3D (11.14%) was significantly higher than T0D, while there was no significant difference among T1D, T2D, T3D. In addition, T0C, T1C, T2C, and T3C showed similar 1000-kernal weight and grain yield.

**Table 1:** Effects of parental drought-priming on grain yield and yield components in offspring under drought stress during grain filling in wheat.

Treatment	Spikes per pot	Kernels per spike	1000-kernel mass (g)	Yield (g pot^-1^)
T0C	20.3 a	47.1 a	42.0 a	40.2 ab
T1C	21.0 a	46.8 a	40.8 a	40.0 ab
T2C	20.7 a	47.6 a	41.2 a	40.4 a
T3C	21.0 a	47.8 a	41.5 a	41.5 a
T0D	20.3 a	47.7 a	34.1 c	33.1 d
T1D	21.3 a	47.1 a	37.6 b	37.8 bc
T2D	20.0 a	48.0 a	38.2 b	36.6 c
T3D	20.3 a	47.8 a	37.9 b	36.8 c


*T0C- no former-generation priming + no offspring drought stress, T0D- no former-generation priming + offspring drought stress; T1C- one-generation priming + no offspring drought stress, T1D- one-generation priming + offspring drought stress; T2C- two-generation priming + no offspring drought stress, T2D- two-generation priming + offspring drought stress; T3C- three-generation priming + no offspring drought stress, T3D- three-generation priming + offspring drought stress. Different lowercase letters indicate the significant difference at *p* < 0.05 level.*

### Plants Water Status, Photosynthesis and Chlorophyll Fluorescence

**FIGURE 2:**
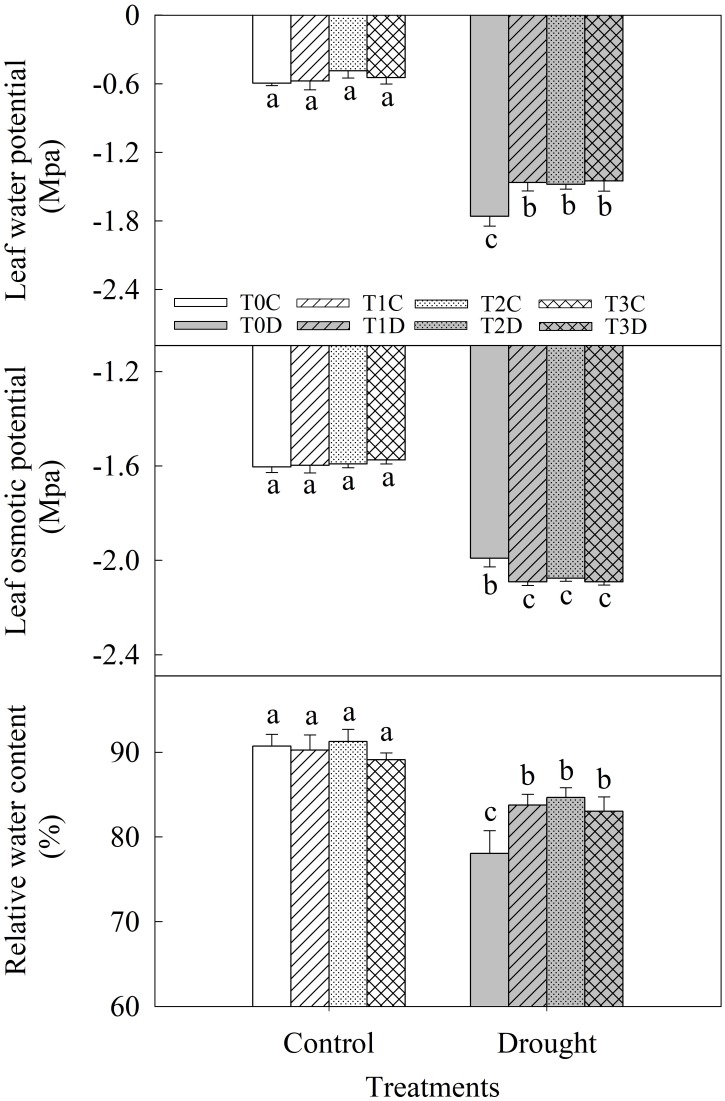
Effects of parental drought-priming on water potential (Ψ_w_), osmotic potential (Ψ_s_) and relative water content (LRWC) of flag leaves of offspring plants under drought stress during grain filling in wheat. T0C, no former-generation priming + no offspring drought stress; T0D, no former-generation priming + offspring drought stress; T1C, one-generation priming + no offspring drought stress; T1D, one-generation priming + offspring drought stress; T2C, two-generation priming + no offspring drought stress; T2D, two-generation priming + offspring drought stress; T3C, three-generation priming + no offspring drought stress; T3D, three-generation priming + offspring drought stress. Data are means ± SE (*n* = 3). Different lowercase letters indicate the significant difference at *p* < 0.05 level.

Drought significantly decreased leaf relative water content (LRWC), predawn water potential (Ψ_w_) and osmotic potential (Ψ_s_) of flag leaves (**Figure 2**). LRWC of T1D, T2D, T3D plants was 7.31, 8.47, and 6.37% higher respectively than the T0D. Ψ_w_ of T1D, T2D, T3D plants were significantly higher while Ψ_s_ was lower than T0D. However, there was no significant difference in these traits among T1D, T2D, and T3D. In addition, there was no difference in water status of flag leaves among T0C, T1C, T2C, and T3C.

**FIGURE 3:**
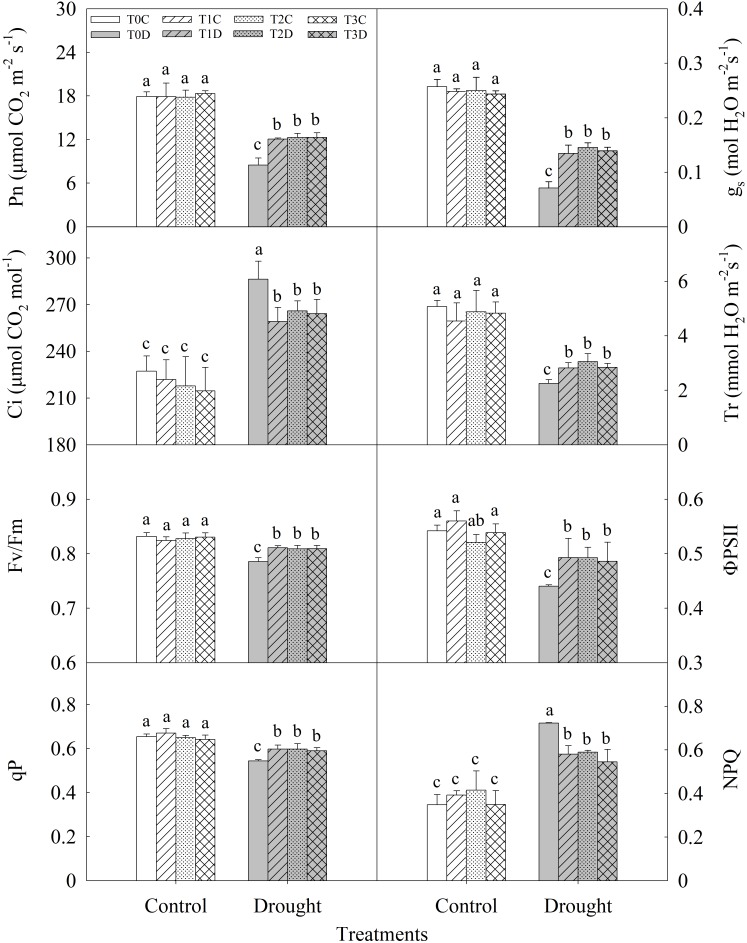
Effects of parental drought-priming on photosynthesis and chlorophyll fluorescence parameters of flag leaves of offspring plants under drought stress during grain filling in wheat. T0C, no former-generation priming + no offspring drought stress; T0D, no former-generation priming + offspring drought stress; T1C, one-generation priming + no offspring drought stress; T1D, one-generation priming + offspring drought stress; T2C, two-generation priming + no offspring drought stress; T2D, two-generation priming + offspring drought stress; T3C, three-generation priming + no offspring drought stress; T3D, three-generation priming + offspring drought stress. Data are means ± SE (*n* = 3). Different lowercase letters indicate the significant difference at *p* < 0.05 level.

There was no significant difference in photosynthesis and chlorophyll fluorescence parameters of flag leaves among T0C, T1C, T2C, and T3C (**Figure 3**). Drought stress significantly decreased net photosynthetic rate (Pn) of flag leaves and T1D (42.32%), T2D (44.82%), T3D (45.10%) plants showed significantly higher Pn as compared with T0D. Stomatal conductance (gs) and transpiration rate (Tr) were significantly higher in T1D, T2D, T3D than in T0D, while intercellular CO_2_ concentration (Ci) was lower. In addition, the maximum quantum efficiency of photosystem II (Fv/Fm) and the actual photochemical efficiency (ΦPSII) were also decreased by drought significantly. T1D, T2D, T3D showed higher Fv/Fm (3.26, 2.98, and 3.02%, respectively) and ΦPSII (11.97, 11.90, and 10.38%, respectively) than T0D. Photochemical quenching of chlorophyll fluorescence (qP) was significantly higher in T1D, T2D, T3D than in T0D, while non-photochemical quenching of chlorophyll fluorescence (NPQ) was lower. Again, there were no significant differences in these parameters among T1D, T2D, and T3D.

### Antioxidant System in Flag Leaves

**FIGURE 4:**
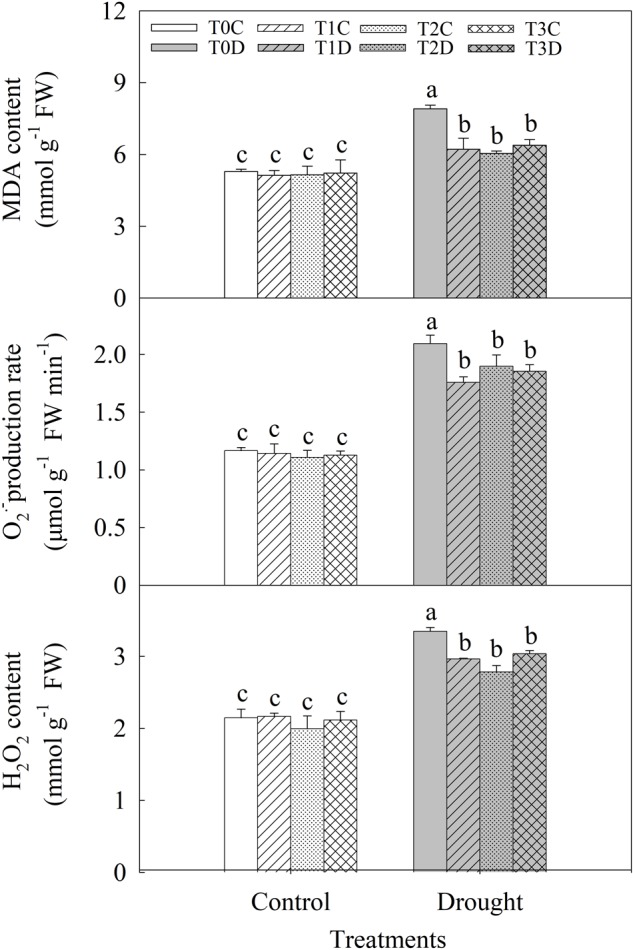
Effects of parental drought-priming on MDA content, $O_{2}^{•-}$ release rate, and H_2_O_2_ content in flag leaves of offspring plants under drought stress during grain filling in wheat. T0C, no former-generation priming + no offspring drought stress; T0D, no former-generation priming + offspring drought stress; T1C, one-generation priming + no offspring drought stress; T1D, one-generation priming + offspring drought stress; T2C, two-generation priming + no offspring drought stress; T2D, two-generation priming + offspring drought stress; T3C, three-generation priming + no offspring drought stress; T3D, three-generation priming + offspring drought stress. Data are means ± SE (*n* = 3). Different lowercase letters indicate the significant difference at *p* < 0.05 level.

Under drought stress, MDA content was significantly increased (**Figure 4**). However, MDA content was much lower in T1D (21.33%), T2D (23.61%), T3D (19.25%) than in T0D. The $O_{2}^{•-}$ release rate and H_2_O_2_ content of flag leaves were significantly increased under drought stress, while they were less affected by drought in the primed (T1D, T2D, T3D) plants than in the non-primed (T0D) plants (**Figure 4**).

Activities of antioxidant enzymes such as SOD, CAT and APX were increased significantly by drought stress and were much higher in the primed (T1D, T2D, T3D) plants than in the non-primed (T0D) plants (**Table 2**). In addition, there was no significant difference in GPX activity among the non-drought treatments and T0D, while it was much higher in the primed (T1D, T2D, T3D) plants under drought.

**Table 2:** Effects of parental drought-priming on function of antioxidant system in flag leaves of offspring plants under drought stress during grain filling in wheat.

Treatments	T0C	T1C	T2C	T3C	T0D	T1D	T2D	T3D
**Activities of enzymes**								
SOD (U mg^-1^ protein)	35.56c	36.50c	35.20c	36.62c	40.01b	46.38a	46.37a	47.66a
CAT (U mg^-1^ protein)	4.22bc	3.97c	4.08bc	4.21bc	4.52b	8.28a	8.42a	8.31a
GPX (U mg^-1^ protein)	0.518b	0.512b	0.525b	0.527b	0.494b	0.688a	0.717a	0.633a
GR (U mg^-1^ protein)	0.555c	0.566c	0.586c	0.559c	0.723b	0.934a	0.893a	0.934a
APX (U mg^-1^ protein)	1.64c	1.66c	1.67c	1.57c	2.24b	4.14a	4.25a	4.71a
MDHAR (U mg^-1^ protein)	4.10a	3.99a	4.07a	4.03a	2.82c	3.37b	3.49b	3.50b
DHAR (U mg^-1^ protein)	0.92c	0.99c	1.04c	0.99c	1.26b	1.39ab	1.51a	1.43ab
**Non-enzymatic antioxidants**								
AsA content (mg g^-1^ FW)	0.380b	0.372b	0.398b	0.377b	0.633a	0.624a	0.641a	0.618a
GSH content (mg g^-1^ FW)	1.02c	1.09c	1.07c	1.08c	1.53b	1.88a	1.79a	1.80a


*T0C, no former-generation priming + no offspring drought stress; T0D, no former-generation priming + offspring drought stress; T1C, one-generation priming + no offspring drought stress; T1D, one-generation priming + offspring drought stress; T2C, two-generation priming + no offspring drought stress; T2D, two-generation priming + offspring drought stress; T3C, three-generation priming + no offspring drought stress; T3D, three-generation priming + offspring drought stress. Different lowercase letters indicate the significant difference at *p* < 0.05 level.*

As for non-enzymatic ROS scavengers, the contents of AsA and GSH increased under drought stress (**Table 2**), moreover, the primed (T1D, T2D, T3D) plants had relatively higher GSH content than the non-primed (T0D) plants. However, there was no significant difference in AsA content among the drought stressed plants and among the control plants (**Table 2**). Activities of GR and DHAR were increased while of MDHAR was decreased by drought, however, they were all higher in the primed (T1D, T2D, T3D) plants than in T0D (**Table 2**). In addition, there was no significant difference in the above-mentioned traits in antioxidant system among T0C, T1C, T2C, and T3C. Gene expression of antioxidant enzymes was also assayed in this study (Supplementary Figure 1), only APX gene expression was consistent with its activity.

### Proline and GB Accumulation

**FIGURE 5:**
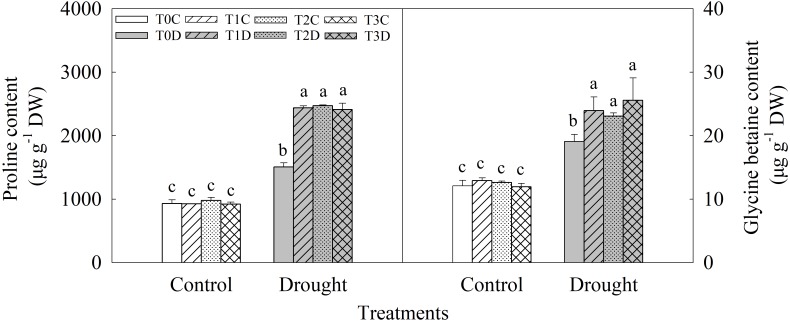
Effects of parental drought-priming on contents of proline and glycine betaine in flag leaves of offspring plants under drought stress during grain filling in wheat. T0C, no former-generation priming + no offspring drought stress; T0D, no former-generation priming + offspring drought stress; T1C, one-generation priming + no offspring drought stress; T1D, one-generation priming + offspring drought stress; T2C, two-generation priming + no offspring drought stress; T2D, two-generation priming + offspring drought stress; T3C, three-generation priming + no offspring drought stress; T3D, three-generation priming + offspring drought stress. Data are means ± SE (*n* = 3). Different lowercase letters indicate the significant difference at *p* < 0.05 level.

Contents of proline and GB significantly increased under drought stress, and the primed (T1D, T2D, and T3D) plants showed higher contents of proline (61.95, 64.07, and 60.11%, respectively) and GB (25.45, 20.80, and 33.89%, respectively) than the non-primed (T0D) plants (**Figure 5**). There was no significant difference among T0C, T1C, T2C, and T3C.

**FIGURE 6:**
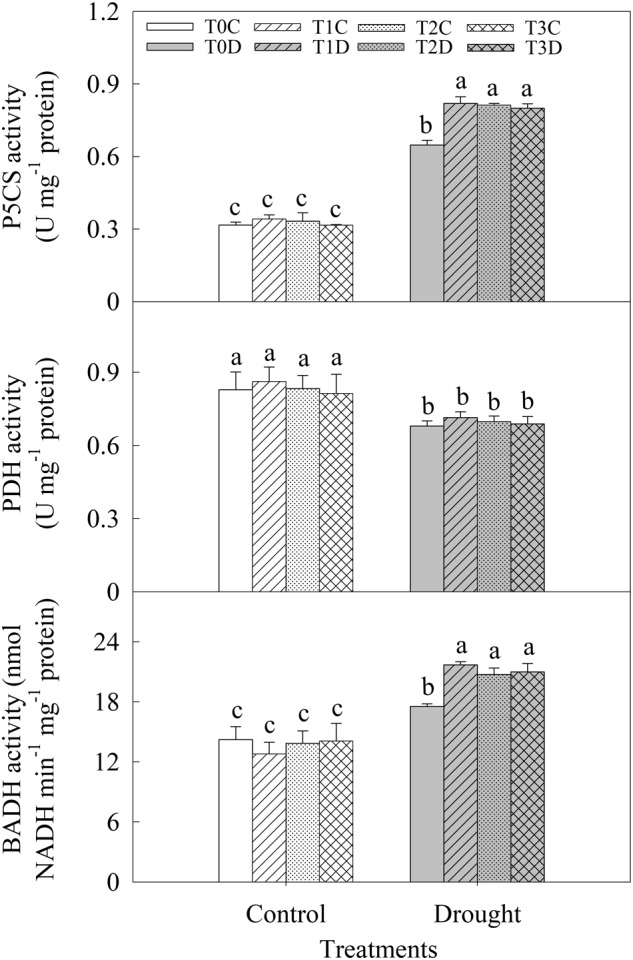
Effects of parental drought-priming on activity of key enzymes involving in proline and glycine betaine metabolism in flag leaves of offspring plants under drought stress during grain filling in wheat. T0C, no former-generation priming + no offspring drought stress; T0D, no former-generation priming + offspring drought stress; T1C, one-generation priming + no offspring drought stress; T1D, one-generation priming + offspring drought stress; T2C, two-generation priming + no offspring drought stress; T2D, two-generation priming + offspring drought stress; T3C, three-generation priming + no offspring drought stress; T3D, three-generation priming + offspring drought stress. Data are means ± SE (*n* = 3). Different lowercase letters indicate the significant difference at *p* < 0.05 level.

Activities of P5CS, PDH, and BADH were measured to reveal the mechanism of accumulations of proline and GB induced by priming (**Figure 6**). Activities of P5CS and BADH were significantly enhanced while PDH activity was decreased due to the drought stress. The primed (T1D, T2D, T3D) plants had relatively higher P5CS (26.54, 25.43, and 23.52%, respectively) and BADH (23.68, 18.21, and 19.73%, respectively) activities than those of the non-primed (T0D) plants under drought. There was no significant difference in PDH activity between the primed (T1D, T2D, T3D) plants and the non-primed (T0D) plants, neither among T0C, T1C, T2C, and T3C plants.

## Discussion

Transgenerational memory, which means the stress events happened during parental generation can be remembered by the plants and pass on to the next generation, and which could help the progeny more effectively to cope with the subsequent stresses (Nosalewicz et al., 2016; Wang et al., 2016b). The mechanisms of transgenerational priming induced tolerance are quite complex, and were reported to be associated with multiple physiological and molecular mechanisms, including epigenetic modifications (chromatin modification, miRNA etc.) (Chinnusamy and Zhu, 2009; Sani et al., 2013; Migicovsky et al., 2014), plant hormone regulation, signaling pathway activation, osmotic adjustment and so on (Hauser et al., 2011; Luna et al., 2012; Zhang et al., 2013). It is not clear whether the drought priming during the former generations could increase drought tolerance in offspring of wheat, and whether the priming responses are different among several successive priming generations. In this study, we imposed wheat plants to three successive generation drought priming and then subjected the offspring plants to drought stress. The results suggested that drought priming during parental generation could induce drought tolerance of offspring in wheat, as exemplified by less reduction grain yield of the primed (T1D, T2D, T3D) plants compared with the non-primed (T0D) plants under drought, which may results from better maintenance of photosynthesis, greater antioxidant capacity and higher osmolytes accumulation in the primed plants.

### Parental Generation Drought Priming Enhances the Osmolytes Accumulation to Maintain Plant Water Status in Offspring Under Drought

Leaf water potential (Ψ_w_) and relative water content (LRWC) are important indicators of plant water status (Flower and Ludlow, 1986). In this study, after 5 days drought stress, Ψ_w_ and LRWC of flag leaves was much lower in relation to the non-stressed plants. However, the primed (T1D, T2D, T3D) plants showed relatively higher LRWC and Ψ_w_ than the non-primed (T0D) plants. The results were in line with our previous studies that drought priming during vegetative growth stages facilitated wheat plants to maintain higher leaf water status than the non-primed plants under drought during grain filling (Wang et al., 2014c). This indicates that parental drought priming could also help to maintain plant water status in offspring exposure to drought stress. Therefore, it is suggested that drought priming in parental plants could enhance drought tolerance in progenies plants.

The higher leaf water status may be owing to the higher accumulation of osmolytes to lower the osmotic potential, and that maintained turgor pressure (Chaves et al., 2003). Proline and GB are two major organic osmolytes and play important roles in plant abiotic stress resistance, such as facilitate plants to retain or absorb water by decreasing the osmotic potential in cells and protect the PSII complex from damage (Ashraf and Foolad, 2007; Wang et al., 2014a). Accumulations of proline and GB were stimulated under drought stress and were significantly higher in tolerant cultivars than sensitive cultivars in wheat (Ashraf and Foolad, 2007; Khoshro et al., 2013). In this study, the primed (T1D, T2D, T3D) plants had higher proline and GB contents than the non-primed (T0D) plants, which was corresponded to significantly lower Ψ_s_ in the primed (T1D, T2D, T3D) plants. This indicates that drought priming in parental plants could decrease Ψ_s_ of flag leaves at least partially via the accumulation of proline and GB hereby maintain turgor pressure and higher LRWC in offspring suffering from drought stress.

Glutamate pathway is the predominant pathway of proline synthesis under osmotic stress in plants (Delauney et al., 1993), in which Δ1-pyrroline-5-carboxylate synthetase (P5CS) reduces glutamate to glutamate-semialdehyde (GSA), GSA then spontaneously converted to pyrroline-5-carboxylate (P5C) which is further reduced to proline by P5C reductase (P5CR). P5CS is the rate limiting enzyme in the process (Hu et al., 1992; Verbruggen et al., 1993). In proline catabolism, proline dehydrogenase (PDH) first convert proline to P5C and P5C then converted to glutamate by P5C dehydrogenase (P5CDH), and *PDH* could be down-regulated by dehydration stress (Kiyosue et al., 1996; Szabados and Savoure, 2010). In drought stressed wheat plants, *P5CS* was significantly induced, in paralleling with increased activity of P5CS and decreased activity of PDH, which promoted the accumulation of proline (Aprile et al., 2009; Khoshro et al., 2013; Jiang et al., 2014). Our results showed that, the activities of P5CS increased and PDH decreased, these could contribute to the accumulation of proline under drought stress. The primed (T1D, T2D, T3D) plants showed higher activity of P5CS, and non-significant difference of PDH activity compared with the non-primed (T0D) plants, indicating that higher proline content induced in the primed plants was mainly ascribed to the elevated P5CS activity rather than modification of PDH activity.

BADH (betaine aldehyde dehydrogenase) is one of the key enzymes for GB biosynthesis (Sakamoto and Murata, 2002). BADH could be induced and showed positive correlation with the GB content under drought stress in wheat (Khoshro et al., 2013). In this study, BADH activity was increased significantly under drought stress and it was higher in the primed (T1D, T2D, T3D) plants than in the non-primed (T0D) plants, which was consistent with the higher GB content in these plants.

### Parental Generation Drought Priming Improves Photosynthesis Performance and Final Yield in Offspring Under Drought

It has been reported that photosynthesis during grain filling stage contributes approximately 70–90% of photo-assimilates to the final grain yield under the favorable conditions (Austin et al., 1977; Bidinger et al., 1977), while post-anthesis photosynthates are highly susceptible to drought (Huseynova et al., 2016). Drought stress significantly restricts photosynthesis through stomatal limitation or non-stomatal limitation (Caemmerer and Farquhar, 1981; Lawlor and Tezara, 2009). In this study, gs significantly decreased and Ci increased by drought stress, suggested that decreased photosynthesis rate was caused by both stomatal and non-stomatal limitation (Farquhar and Sharkey, 1982). However, the parentally primed (T1D, T2D, T3D) plants showed higher Pn and gs as well as lower Ci than T0D, suggesting that there was less inhibition of stomatal and non-stomatal factors in the primed plants. Fv/Fm, ΦPSII and qP, which can reflect the capacity of photosystem II, can be reduced by severe drought stress (Maxwell and Johnson, 2000; Wang et al., 2016a). Here, the higher Fv/Fm, ΦPSII and qP values as well as lower NPQ observed in the primed (T1D, T2D, T3D) plants indicates that parental drought priming contributed to maintain relatively higher potential quantum efficiency, electron transport rate and the actual photosynthetic ability of PSII in offspring of wheat under drought stress, which is in accordance with higher Pn in the primed plants.

Grain yield data in the present study was consistent with photosynthesis, and the impact of drought on grain yield was mainly ascribed to the decline of 1000-kernal weight since the drought stress was applied during the grain filling stage. 1000-kernal weight yield of the primed (T1D, T2D, T3D) plants was higher than the non-primed (T0D) plants. This is in line with priming effect during seedling and stem elongation in our previous study (Wang et al., 2014b). Since the post-anthesis photoassimilates contribute the most part of grain filling (Austin et al., 1977; Bidinger et al., 1977), the higher grain weight and final yield of the parentally primed (T1D, T2D, T3D) plants under drought treatment at least could be partially explained by the higher maintenance of the higher photosynthesis capacity than the non-primed (T0D) plants.

### Parental Generation Drought Priming Contributes to Alleviate Oxidative Stress Damage in Offspring Under Drought

The inhibition of photosynthesis could lead to a higher leakage of electrons to O_2_ by the Mehler reaction facilitating the production of a large amount of ROS under drought in wheat (Cruz de Carvalho, 2008). ROS would further cause lipid peroxidation, MDA production and ultimately result in cell damage and plant death (Cruz de Carvalho, 2008). In this study, we observed that MDA content was increased significantly by drought stress. However, it was much lower in the primed (T1D, T2D, T3D) plants than in the non-primed (T0D) plants. In line with MDA content, $O_{2}^{•-}$ release rate and H_2_O_2_ content of flag leaves were significantly increased under drought, while they were also less produced in the primed (T1D, T2D, T3D) plants than in the non-primed (T0D) plants.

Superoxide dismutase can dismutate superoxide to H_2_O_2_, acting as the first line of defense against ROS. Subsequently, CAT, APX and GPX were activated to detoxify H_2_O_2_ (Apel and Hirt, 2004). Antioxidant enzymes increased significantly under drought stress in wheat and the better drought tolerance performance in the tolerant cultivar was related to its higher antioxidant enzymes activities than the sensitive cultivar (Zhang and Kirkham, 1994; Sairam and Saxena, 2000). In this study, drought stress significantly increased activities of SOD, CAT and APX to cope with increased $O_{2}^{•-}$ release rate and H_2_O_2_ content. Moreover, they were all higher in the primed (T1D, T2D, T3D) plants than in the non-primed (T0D) plants. For GPX activity, there was no significant difference among the non-drought treatments and T0D, while it was much higher in the primed (T1D, T2D, T3D) plants under drought. For other enzymes involving in A-G cycle and GPX cycle, activities of MDHAR, DHAR, and GR were all higher in the primed (T1D, T2D, T3D) plants than in T0D. As for non-enzymatic ROS scavenging mechanisms, AsA and GSH could be oxidized by H_2_O_2_ so as to degrade H_2_O_2_ (Apel and Hirt, 2004). In this study, the contents of AsA and GSH increased by drought stress, and the primed (T1D, T2D, T3D) plants showed higher GSH content than the non-primed (T0D) plants. There was no significant difference in AsA content under drought. The increased activities of enzymes and higher GSH content contributed to the lower $O_{2}^{•-}$ release rate, H_2_O_2_ content and MDA content of flag leaves in the primed (T1D, T2D, T3D) plants than in the non-primed (T0D) plants. The above results illustrate that drought priming in parental plants could induce up-regulation of the antioxidant defense system in both enzymatic and non-enzymatic approaches to alleviate oxidative damage in offspring plants.

Research has shown that the induction of transcripts that encode antioxidant enzymes plays critical roles in cellular redox homeostasis (Dutilleul et al., 2003). Thus, gene expression of antioxidant enzymes was also measured in this study. Transcript levels of *CAT, APX, GPX, GR, MDHAR* and *DHAR* were all increased under drought stress, consistent with enzymes activities except MDHAR. However, only *APX* showed higher transcript levels in the primed (T1D, T2D, T3D) plants than in the non-primed (T0D) plants. Therefore, higher activities of antioxidant enzymes in the primed (T1D, T2D, T3D) plants may be results of other explanations rather than the up-regulation of their encoding genes.

It has been proved that proline and GB play multiple functions in plants under stress, such as stabilizes the redox balance in photosystem, scavenges ROS; protects protein stability and enhances the activities of antioxidant enzymes (Chaves et al., 2003; Szabados and Savoure, 2010). In accordance with this, the increased accumulation of proline and GB in the primed (T1D, T2D, T3D) plants may contribute to protect PSII and alleviate damages from drought to PSII, resulting in higher photosynthetic capacity as well as less production of ROS. Moreover, enhanced proline production could scavenge more ROS and contribute to a higher maintenance of activities of antioxidant enzymes discussed above.

Ding et al. (2012) found that the transcription rate and transcript levels of a subset of dehydration-response genes were increased significantly during recurring dehydration stresses in the same generation in *Arabidopsis thaliana*, which was then defined as “transcriptional memory” and this kind of genes were named as “trainable genes.” Luna et al. (2012) reported that progeny from bacteria-inoculated Arabidopsis (P1) were primed to activate salicylic acid-inducible defense genes of which promoters had changed histone modifications and were more resistant to the bacteria infection reoccurring again. There are few researches to investigate the effects of drought stress priming other than dehydration stress on the stress tolerance of the next generation other than the same generation in wheat. In this study, the elevated activities of P5CS and BADH in the parentally primed (T1D, T2D, T3D) plants may be resulted from up-regulation of *P5CS* and *BADH* expression, *P5CS* and *BADH* may be “trainable genes” playing roles in the transgenerational drought stress memory, or they could be regulated by the upstream signals which related genes have been trained. The “memory” factors should be changes of epigenetic modifications such as DNA methylation and histone modifications in “trainable genes.” In addition, ABA plays important roles in priming induced drought stress tolerance (Wang et al., 2014c) and cold stress tolerance (Li et al., 2015) in the same generation, and the roles and mechanisms of ABA in priming induced transgenerational stress tolerance are far from clear and need further study.

It is interesting that there was no difference in all of the traits measured in this study among the primed (T1C, T2C, T3C) plants and non-primed (T0C) plants under non-drought conditions. This suggested that the parentally drought primed plants did not affect offspring plants in the physiological level under non-drought conditions, which was consistent with our previous finding (Wang et al., 2016b; Zhang et al., 2016). Furthermore, there was no significant difference in most traits we measured among three generations of the parentally primed (T1D, T2D, T3D) plants under drought stress. Research in *Arabidopsis thaliana* has shown that transgenerational memory may be unstable and occur in a stochastic manner (Pecinka et al., 2009; Lang-Mladek et al., 2010). Therefore the improvement in drought tolerance by priming among the three generations is not suggested as a simple additive effect.

## Conclusion

**FIGURE 7:**
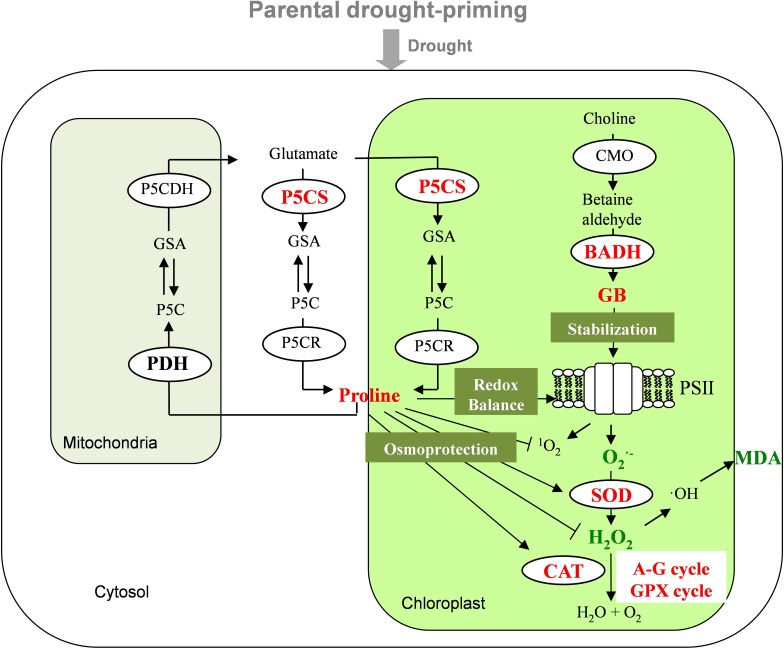
Mechanisms of parental drought-priming enhances tolerance to post-anthesis drought in offspring of wheat. The abbreviations of the enzymes or processes in bold were those included in the study, those in red, green and black indicate being up-regulated, down-regulated and not affected by the priming and stress, respectively. The parental drought-priming induces up-regulation of P5CS and BADH activities, which promotes the accumulation of proline and GB in offspring plants under the post-anthesis drought stress. Proline contributes to maintain leaf water status, stabilize the redox balance in photosystem, and enhance the activities of antioxidant enzymes (SOD, CAT, and enzymes in A-G cycle and GPX cycle), so as to decrease the production of MDA and then protect protein stability. And enhanced accumulation of GB could alleviate damages to PSII through accelerating D1 protein turnover to alleviate the photo-damage under drought.

In conclusion, drought priming in parental plants could induce transgenerational stress tolerance to drought in offspring. Under drought stress, the parentally primed plants elevated activities of P5CS and BADH, which contributed to the enhanced accumulation of proline and GB. The accumulation of proline and GB played critical roles in osmotic adjustment to maintain higher plant water status, and also may contribute to less inhibition of photosynthesis, and higher ROS scavenging capacity (**Figure 7**). Therefore, the enhanced accumulation of proline and GB could play critical roles in parental priming induced alleviation of the drought damages. There was no significant difference in the alleviation effects on drought stress induced by different generations of priming, implying that one generation’s priming is enough to improve the tolerance of offspring plants to drought stress. In addition, the parental drought priming had no significant effect on offspring in terms of physiological processes and grain yield under non-drought conditions. This infers a potential approach to cope with the unpredicted drought stress by parental abiotic stress priming without side effect if the drought stress does not occur.

## Author Contributions

DJ and XW designed the experiments. XlW, XZ, and JCh performed the experiments and data analysis. XlW, XW, QZ, JCa, TD, WC, and DJ involved in the results discussion, manuscript writing and revising. All authors have read and approved the final manuscript.

## Conflict of Interest Statement

The authors declare that the research was conducted in the absence of any commercial or financial relationships that could be construed as a potential conflict of interest.
