# Supplementary material for: Parental Drought-Priming Enhances Tolerance to Post-anthesis Drought in Offspring of Wheat
Source: Front Plant Sci. 2018 Mar 1;9:261. doi: 10.3389/fpls.2018.00261 (PMC5838469; doi:10.3389/fpls.2018.00261)
Supplement: Supplementary file 1 [file Table_1.docx]

Supplementary Material

Parental drought-priming enhances tolerance to post-anthesis drought in offspring of wheat

Xiulin Wang, Xiaxiang Zhang, Jing Chen, Xiao Wang*, Jian Cai, Qin Zhou, Tingbo Dai, Weixing Cao, Dong Jiang*

*** Correspondence:** Xiao Wang: [xiaowang@njau.edu.cn](mailto:xiaowang@njau.edu.cn) ; Dong Jiang: [jiangd@njau.edu.cn](mailto:jiangd@njau.edu.cn)

# Supplementary Figures and Tables

## Supplementary Figures

Relative expression of *MDHAR*

Relative expression of *GR*

Relative expression of *CAT*

Relative expression of *Cu/Zn-SOD*

Relative expression of *GPX1*

Relative expression of *DHAR*

Relative expression of *APX*

Relative expression of *Mn-SOD*

Treatments

**Supplementary Figure 1** Effects of parental drought-priming on relative expression of genes coding enzymes in antioxidant system of flag leaves in offspring plants under drought stress during grain filling in wheat. T0C- no former-generation priming + no offspring drought stress, T0D- no former-generation priming + offspring drought stress; T1C- one-generation priming + no offspring drought stress, T1D- one-generation priming + offspring drought stress; T2C- two-generation priming + no offspring drought stress, T2D- two-generation priming + offspring drought stress; T3C- three-generation priming + no offspring drought stress, T3D- three-generation priming + offspring drought stress. Data are means ± SE (n = 3). Different lowercase letters indicate the significant difference at p < 0.05 level.

## Supplementary Tables

**Supplementary Table 1** Leaf relative water content of parent plants after drought priming and the effect of drought on 1000-kernel weight of the parent plants. ‘P’ and ‘N’ indicate the drought priming and non-priming during the first three-year’s pretreatment, respectively. ‘n’ and ‘p’ in the brackets indicate the non-primed and primed parental plants, respectively. The number of ‘n’ or ‘p’ indicates the years of being treated. For instance, n, nn, nnn indicate the non-primed parental plants in the first, the second and the third year, respectively, while p, pp, ppp indicate the primed parental plants in the first, the second and the third year, respectively. Similarly, np and pp indicate non-priming in the first year with priming in the second year, and priming in both first and second year, respectively; nnp and npp indicate non-priming in the first and second year with priming in the third year, and non-priming in the first year with priming in the second and third year, respectively. The different superscripted lowercase letters indicate the significant difference at p < 0.05 level.

| Traits | Growth season | | | | | | | |
| --- | --- | --- | --- | --- | --- | --- | --- | --- |
|  | 1^st^ year | |  | 2^nd^ year | |  | 3^rd^ year | |
|  | P | N |  | P | N |  | P | N |
| Leaf relative water content (%) |  | 90.7^a^ (n） |  |  | 91.2^a^ (nn） |  |  | 90.3^a^ (nnn) |
|  |  |  |  |  |  |  | 85.1^b^ (nnp) |  |
|  |  |  |  | 85.9^b^ (np) |  |  | 86.4^b^ (npp) |  |
|  | 85.5^b^ (p） |  |  | 86.5^b^ (pp) |  |  | 86.1^b^ (ppp) |  |
| 1000-kernel weight (g) |  | 38.9^a^ (n） |  |  | 39.4^a^ (nn) |  |  | 38.5^a^ (nnn) |
|  |  |  |  |  |  |  | 36.6^b^ (nnp) |  |
|  |  |  |  | 35.9^b^ (np) |  |  | 36.9^b^ (npp) |  |
|  | 36.2^b^ (p） |  |  | 36.1^b^ (pp) |  |  | 37.2^b^ (ppp) |  |

**Supplementary Table 2** Sequences and its source of all primers used in Quantitative Real-time PCR. F- forward primer; R- reverse primer.

| Gene | Sequence 5'- 3' | Product size (bp) | Source |
| --- | --- | --- | --- |
| *Mn-SOD* | F: CCGGACTACCTGACCAACATC  R: CCAACAGCGGGAAACTCAA | 135 | ([Wang et al., 2011](#_ENREF_3)) |
| *Cu/Zn-*  *SOD* | F: TGGGAGAGCGTTTGTTGTTC  R: GTCTTCCACCAGCATTTCCA | 92 | ([Wang et al., 2011](#_ENREF_3)) |
| *CAT* | F: CCATGAGATCAAGGCCATCT  R: ATCTTACATGCTCGGCTTGG | 103 | ([Wang et al., 2011](#_ENREF_3)) |
| *GPX1* | F: CTCGCTTCAAGGCTGAGTA  R: CCACCTTTGCTAGACTTCAG | 97 | ([Wei et al., 2015](#_ENREF_4)) |
| *GR* | F: TGCGTCCCGAAGAAGATACT  R: GTTGATGTCCCCGTTGATCT | 96 | ([Wang et al., 2011](#_ENREF_3)) |
| *APX* | F:AAAACCACCTACTGCCACCCTATC  R: AGCATTCGCTCCATGACTCAACT | 148 | ([Qiu et al., 2014](#_ENREF_2)) |
| *MDHAR* | F: AGAAGTTTACGCCCTTCGGC  R: TTGGAATGTCATCGCCATC | 132 | ([Wei et al., 2015](#_ENREF_4)) |
| *DHAR* | F: GTGCCTGTGTATAACGGTG  R: ACAAGTGATGGAGTTGGGT | 94 | ([Wei et al., 2015](#_ENREF_4)) |
| *ADP-RF* | F: GCTCTCCAACAACATTGCCAAC  R: GCTTCTGCCTGTCACATACGC | 165 | ([Paolacci et al., 2009](#_ENREF_1)) |

# REFERENCES

Paolacci, A.R., Tanzarella, O.A., Porceddu, E., Ciaffi, M., 2009. Identification and validation of reference genes for quantitative RT-PCR normalization in wheat. BMC molecular biology 10, 11.

Qiu, Z., Guo, J., Zhu, A., Zhang, L., Zhang, M., 2014. Exogenous jasmonic acid can enhance tolerance of wheat seedlings to salt stress. Ecotoxicology and environmental safety 104, 202-208.

Wang, X., Cai, J., Jiang, D., Liu, F., Dai, T., Cao, W., 2011. Pre-anthesis high-temperature acclimation alleviates damage to the flag leaf caused by post-anthesis heat stress in wheat. Journal of plant physiology 168, 585-593.

Wei, L., Wang, L., Yang, Y., Wang, P., Guo, T., Kang, G., 2015. Abscisic acid enhances tolerance of wheat seedlings to drought and regulates transcript levels of genes encoding ascorbate-glutathione biosynthesis. Frontiers in plant science 6, 458.
